# Ring-shaped meniscus formation may arise from the regenerative response to meniscectomy during growth spurts: A case report

**DOI:** 10.1016/j.ijscr.2019.07.056

**Published:** 2019-07-25

**Authors:** Masaki Nagashima, Toshiro Otani, Kota Kojima, Shinsuke Aida, Ken Ishii

**Affiliations:** a Department of Orthopaedic Surgery, International University of Health and Welfare Mita Hospital, 1-4-3, Mita, Minato-ku, Tokyo, 108-8329, Japan; b Department of Orthopaedic Surgery, School of Medicine, International University of Health and Welfare, 4-3 Kōzunomori, Narita city, Chiba, 286-8686, Japan; c Institute for Integrated Sports Medicine Keio University School of Medicine, 35 Shinanomachi, Shinjuku-ku, Tokyo, 160-8582, Japan; d Department of Pathology, International University of Health and Welfare Mita Hospital, 1-4-3, Mita, Minato-ku, Tokyo, 108-8329, Japan

**Keywords:** Ring-shaped meniscus, Discoid meniscus, Knee, Arthroscopic meniscectomy, Case report

## Abstract

**Introduction:**

Ring-shaped meniscus is very rare and generally considered to be a congenital malformation. Here, we report a case of newly formed ring-shaped meniscus-like tissue following arthroscopic partial meniscectomy for a complete discoid lateral meniscus.

**Presentation of case:**

A 14-year-old boy underwent arthroscopic partial meniscectomy for complete discoid lateral meniscus of the left knee. The growth plates were present at the surgery. Although pain improved immediately after surgery, he reported left knee pain at the 7-month follow-up. Magnetic resonance imaging revealed a bucket-handle tear of the lateral meniscus and a second arthroscopic surgery was performed. During arthroscopy, we found that the anterior and posterior horns of the lateral meniscus were connected by meniscus-like tissue forming a ring-shaped meniscus and the newly formed interhorn meniscal bridge was resected. Histological evaluation indicated that the newly formed tissue was meniscus-like tissue that had appeared secondary to tissue repair. He visited our hospital at 16 years old, after the growth plates had closed, complaining of right knee pain. Arthroscopic partial meniscectomy was performed for complete discoid lateral meniscus of the right knee. As of two years after this surgery, no morphological changes have been observed.

**Discussion:**

Although the exact mechanisms of meniscus-like tissue formation remain unclear, the growth spurt may promote the mechanisms of tissue repair, forming ring-shaped meniscus-like tissue.

**Conclusion:**

This is the first case of ring-shaped meniscus-like tissue forming after partial resection of a complete discoid lateral meniscus. Ring-shaped meniscus-like tissue can develop in growing patients.

## Highlights

• We presented a case of newly formed ring-shaped meniscus-like tissue after partial resection of discoid lateral meniscus.
• The newly formed interhorn bridge could have represented a meniscus-like tissue that appeared secondary to tissue repair.
• Intra-patient findings indicate that growth spurts may promote the tissue repair.

## 1 Introduction

Several morphological anomalies of the lateral meniscus exist, including discoid meniscus, double-layer meniscus, hypoplastic meniscus, and ring-shaped meniscus. Of these, the most common anomaly is discoid meniscus. Ring-shaped meniscus is relatively rare, with an incidence of 0.9% to 2.4% [1,2] and has been proposed as a fourth variant of lateral meniscus abnormalities in the Watanabe classification [3,4]. Although ring-shaped meniscus is generally considered a congenital malformation [[4], [5], [6], [7]], degenerative alteration from chronic bucket handle tears or central perforation of complete discoid and regeneration of the meniscus-like tissue have been reported as possible causes of this abnormality [8,9]. Soejima et al. [8] and Fujii et al. [9] reported cases in which they had identified ring-shaped meniscal regeneration following anterior cruciate ligament (ACL) reconstruction. They speculated that bone marrow mesenchymal stem cells derived from the bone tunnels or metaplasia of the ACL remnants caused such malformations. To the best of our knowledge, no reports have described regenerated ring-shaped meniscus following arthroscopic meniscectomy without bone or ligament treatments. Here, we report the case of newly formed ring-shaped meniscus-like tissue following arthroscopic partial meniscectomy for complete discoid lateral meniscus. This work has been reported in line with the SCARE criteria [10].

## 2 Presentation of case

**Fig. 1:**
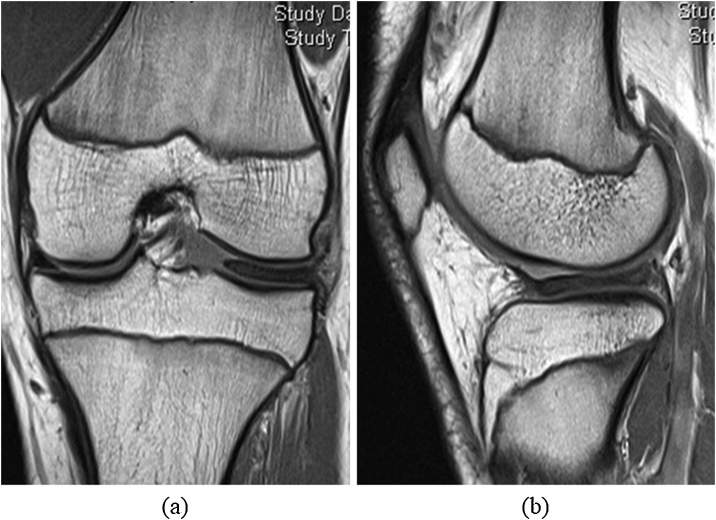
MR images before first arthroscopic surgery for the left knee. Coronal (a) and sagittal (b) proton density-weighted images show a complete discoid lateral meniscus. Growth plates are visible.

**Fig. 2:**
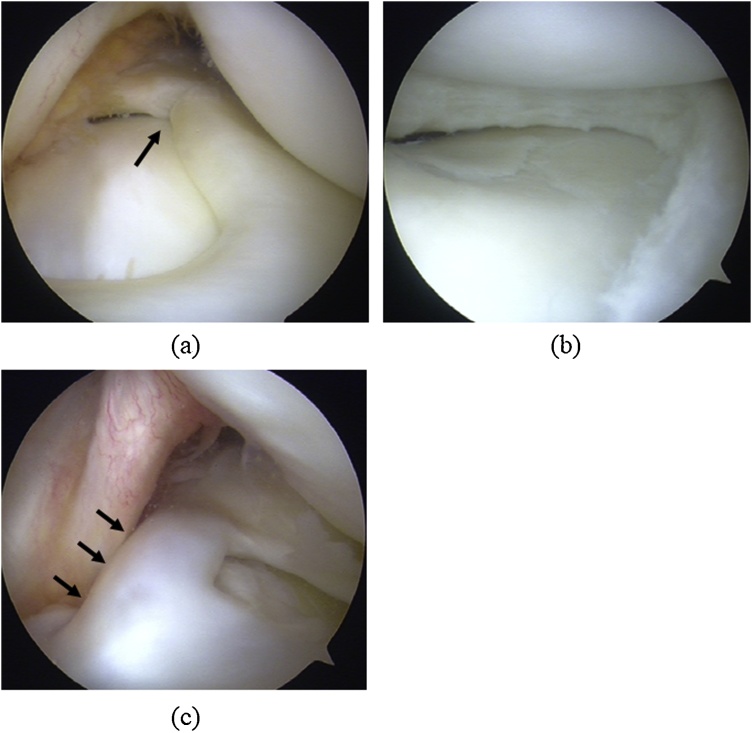
Arthroscopic findings. Complete discoid lateral meniscus with radial tear (a: black arrow) was treated by partial meniscectomy (b). Images from re-arthroscopy 7 months after the first arthroscopic surgery reveal that the anterior and posterior horns of the lateral meniscus are connected by meniscus-like tissue to resemble a ring-shaped meniscus (c: black arrows).

A 14-year-old boy visited our hospital complaining of worsening left knee pain. He had twisted the left knee while playing basketball 18 months earlier. On physical examination, range of motion in the left knee was 0–140°. The patient showed tenderness on palpation along the lateral aspect of the joint line and a positive result for McMurray’s test on the lateral side. X-rays revealed that growth plates on both the femur and tibia were still present. Magnetic resonance imaging (MRI) revealed a complete lateral discoid meniscus with degeneration within the meniscus (Fig. 1). The clinical diagnosis was complete lateral discoid meniscus, and the first arthroscopic surgery was performed. Complete lateral discoid meniscus with a radial tear was confirmed on arthroscopic examination (Fig. 2a), and partial meniscectomy was performed (Fig. 2b). No other abnormalities were noted. Partial to full weight-bearing was allowed as tolerated without immobilization of the knee, and physiotherapy including active motion exercises and thigh muscle strengthening started on postoperative day 1. After arthroscopic surgery, pain in the left knee improved, and the patient returned to playing basketball 5 months after the surgery.

At the 7-month follow-up, the patient revisited our hospital complaining of left knee pain and left knee locking developed during daily activity. No history of recent trauma was elicited. His height had increased 2.4 cm since the first surgery. On physical examination, range of motion in the left knee was 0–130°, with a positive result for McMurray’s test of the lateral side. MRI revealed a low-signal cord between the anterior and posterior portions of the lateral meniscus, resembling a bucket handle tear of the lateral meniscus. A second arthroscopic surgery was performed based on these findings. During arthroscopy, we found that the anterior and posterior horns of the lateral meniscus were connected by meniscus-like tissue forming a ring-shaped meniscus (Fig. 2c). The newly formed interhorn meniscal bridge had not been present at the first surgery, and was thus suspected to represent the cause of the new knee pain. As a result, this bridge was resected. The posterior potion of the lateral meniscus was sutured with FAST-FIX360 (Smith & Nephew, Andover, MA) after identification of a longitudinal tear with instability. Full weight-bearing was started immediately, but knee flexion was limited to 90° for the first 3 weeks. Six months after the second arthroscopic surgery, the patient returned to playing basketball without any symptoms.

**Fig. 3:**
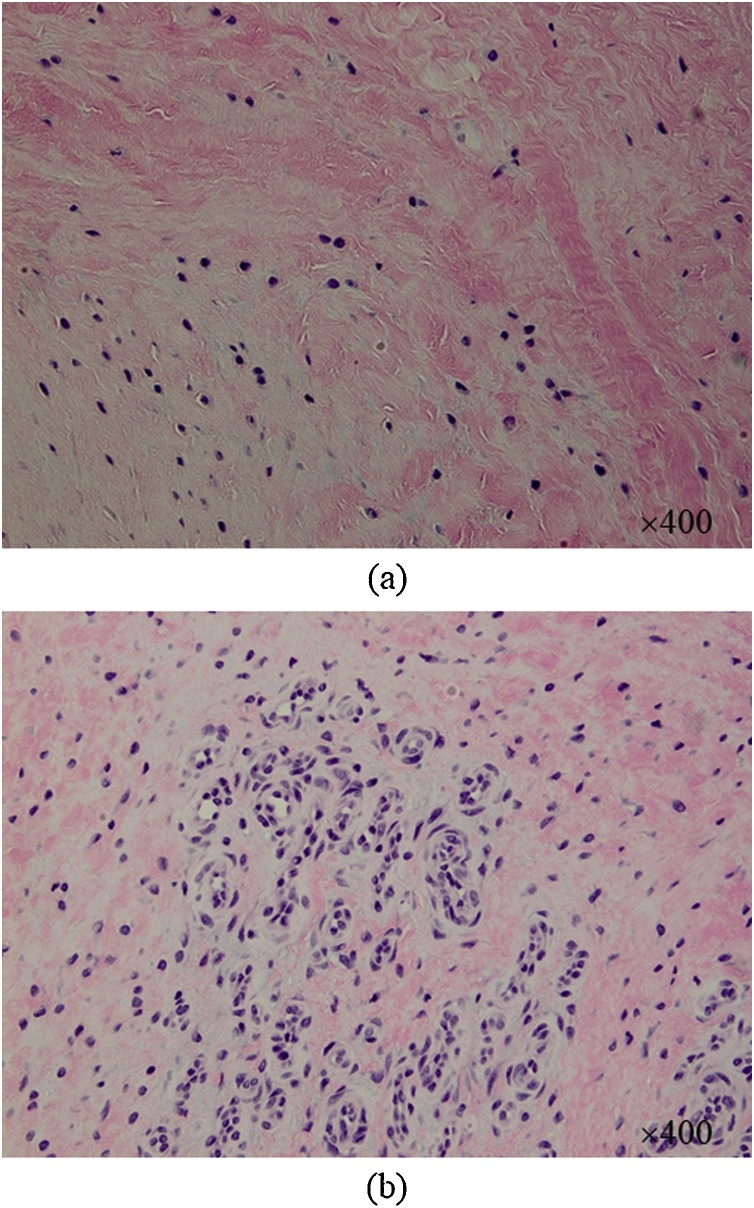
Histologic findings for the newly formed interhorn meniscal bridge. Hematoxylin and eosin staining shows areas of chondrocyte-like cells mixed in collagenous fibers (a), and capillary vessels infiltrating the region (b), indicating the newly formed interhorn meniscal bridge comprises meniscus-like tissue appearing secondary to tissue repair.

Hematoxylin and eosin staining of the newly formed interhorn meniscal bridge showed areas of chondrocyte-like cells mixed in collagenous fibers (Fig. 3a), with capillary vessels infiltrating the region (Fig. 3b). The newly formed interhorn bridge could thus have represented a meniscus-like tissue that appeared secondary to tissue repair.

One year after the second arthroscopic surgery, at 16 years old, the patient visited our hospital with right knee pain after stumbling on some stairs. Although the growth plates on both femur and tibia had closed, physical examination and MRI indicated complete discoid lateral meniscus similar to that in the left knee. Arthroscopic partial meniscectomy was performed. Currently, as of two years after the right knee surgery and three years after the second surgery on the left knee, the patient remains asymptomatic. Recent follow-up MRI of both knees has not shown any morphological changes.

Written informed consent was obtained from the patient and his family for publication of this case report and accompanying images.

## 3 Discussion

Ring-shaped lateral meniscus is a rare anomaly, and characterized by a ring-shaped morphology with normal posterior tibial attachment [4]. Ryu et al. [1] reported a ring-shaped lateral meniscus among 4 of 437 knees (0.9%) in a cadaveric study. Kim et al. [2] reported arthroscopic findings for 164 of 1221 knees (13.4%) showing morphologic malformations of the lateral meniscus. Among these 164 knees, 4 knees (2.4%) showed ring-shaped lateral menisci. Noble et al. [5] reported the first case of congenital ring-shaped lateral meniscus, associated with congenital absence of the ACL. Monllau et al. [4] reported the first case of congenital ring-shaped lateral meniscus without any other associated malformation. Ring-shaped meniscus is generally considered to be a congenital malformation. Lateral meniscus of some primates is reported to be ring-shaped [11]. However, degenerative alteration and regeneration of the meniscus have been reported as a possible cause [8,9]. Soejima et al. [8] reported a case of regenerated ring-shaped meniscus-like tissue following partial resection of a discoid lateral meniscus and ACL reconstruction. Fujii et al. [9] likewise reported a case of newly formed ring-shaped meniscus-like tissue after ACL reconstruction without partial meniscectomy. They suggested bone marrow mesenchymal stem cells derived from the bone tunnel and metaplasia of the ACL remnants as a possible cause of this regeneration.

In our case, ring-shaped meniscus-like tissue formed after partial resection of a complete discoid lateral meniscus without bone or ligament treatments. To the best of our knowledge, this is the first report of ring-shaped meniscus-like tissue forming after partial resection of a complete discoid lateral meniscus alone.

Although the exact mechanisms remain unclear, formation of this tissue may have been influenced by the growth spurt. Bisicchia et al. [12] reported re-growth of an incomplete discoid lateral meniscus after arthroscopic partial resection in an 11-year-old boy, and discussed whether the growth spurt might have affected meniscal regeneration. In our case, growth plates on both the femur and tibia were present at the first left knee surgery, indicating that he was still growing. Histological evaluation revealed newly formed meniscus-like tissue had regenerated through tissue repair. The growth spurt could potentially have promoted the mechanisms of tissue repair, forming a ring-shaped meniscus-like tissue. Interestingly, when the patient was 16 years old and the growth plates had closed, no meniscal regeneration was replicated after right knee arthroscopic surgery.

For adolescent patients with discoid lateral meniscus that need meniscectomy, it is important to reduce the risk of osteoarthritis progression as much as possible. In symptomatic patients in whom nonsurgical treatment fails, arthroscopic management is indicated [13]. Since long-term results after total meniscectomy revealed high rate of osteoarthritic changes [14], reshaping that preserved peripheral rim of the meniscus is the preferred treatment. Additionally, for the reshaped meniscus with peripheral instability, stabilization with suture repair is necessary [13]. These techniques were reported to have good clinical results [15,16]. However, reshaping cannot completely prevent degenerative changes, and smaller residual meniscal width and meniscal extrusion were significantly correlated with degenerative changes [17]. To prevent osteoarthritis progression after the reshaping, further studies are needed.

## 4 Conclusion

We presented a case of newly formed ring-shaped meniscus-like tissue after partial resection of complete discoid lateral meniscus. Intra-patient findings indicate that growth spurts may promote tissue repair and aid in the formation of meniscus-like tissue. Patients who have been diagnosed with ring-shaped menisci may have experienced an injury during adolescence that caused regeneration of meniscus-like tissue to form an interhorn meniscal bridge.

## Sources of funding

We have no sources of funding for our research.

## Ethical approval

Since our article is a case report, no approval from the Ethics Committee is required in our institution.

## Consent

Written informed consent was obtained from the patient and his family for publication of this case report and accompanying images.

## Author contribution

Masaki Nagashima performed arthroscopic surgery, collected data, and wrote the paper.

Toshiro Otani assisted the arthroscopic surgery and helped to write the paper.

Kota Kojima assisted the arthroscopic surgery and helped to write the paper.

Shinsuke Aida conducted a histological examination.

Ken Ishii revised the paper.

## Registration of research studies

Name of the registry: Research Registry.

UIN: researchregistry4843.

## Guarantor

Masaki Nagashima and Ken Ishii.

## Provenance and peer review

Not commissioned, externally peer-reviewed.

## Declaration of Competing Interest

We have no financial and personal relationships with other people or organisations that could inappropriately influence our work.
